# Supplementary material for: Inactivating hepatitis C virus in donor lungs using light therapies during normothermic ex vivo lung perfusion
Source: Nat Commun. 2019 Jan 29;10:481. doi: 10.1038/s41467-018-08261-z (PMC6351537; doi:10.1038/s41467-018-08261-z)
Supplement: Supplementary file 1 — Supplementary Information [file 41467_2018_8261_MOESM1_ESM.pdf]

A

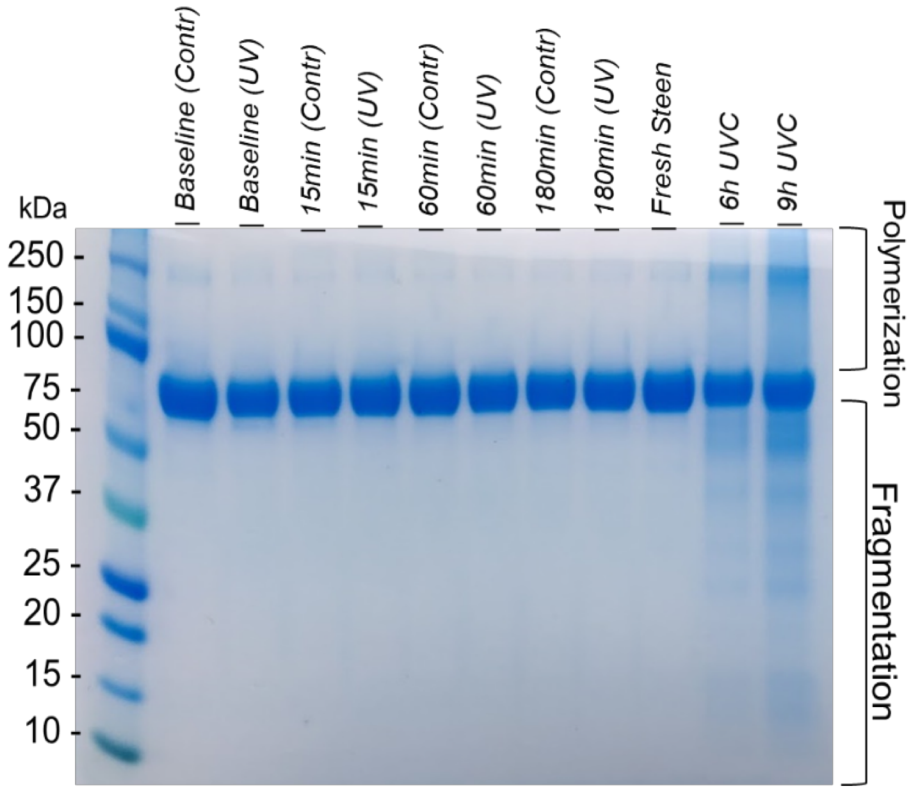

B

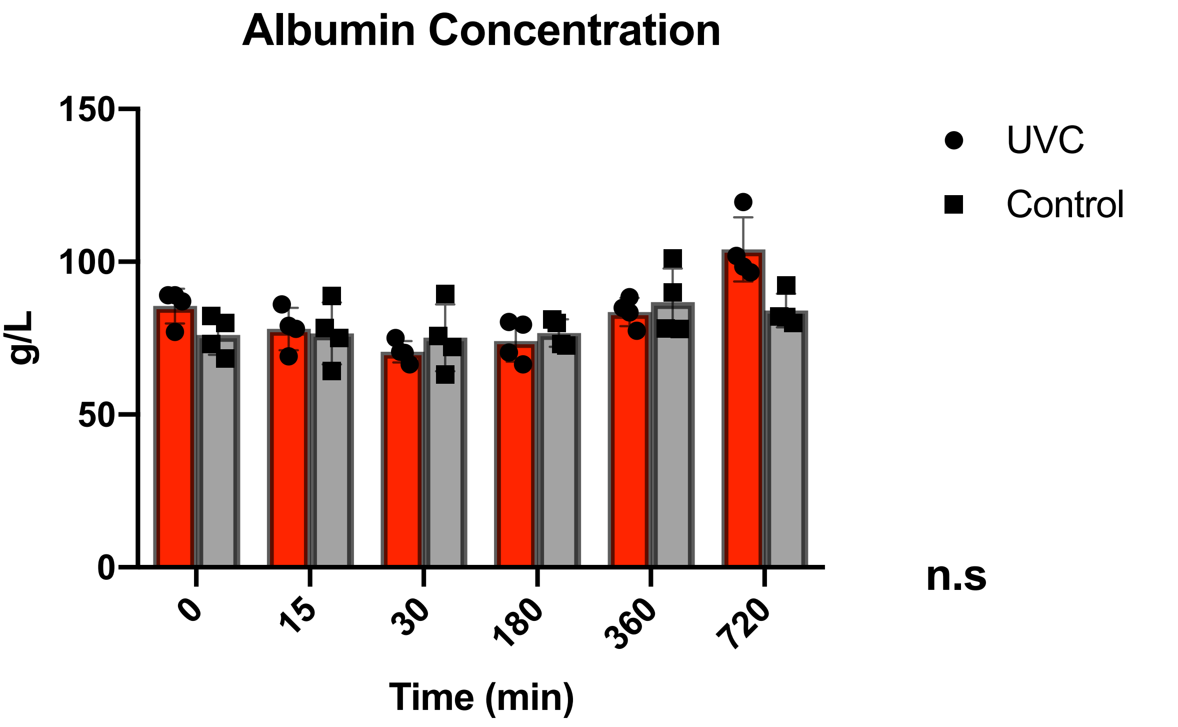

Supplementary Figure 1. Albumin fragmentation and concentration in Steen solution after UVC exposure. (A) No molecular transformation after UVC irradiation was seen until 6h of irradiation, demonstrated by stable molecular weight assessed by a human albumin gel electrophoresis assay. After 6h, light-induced molecular transformation was seen, demonstrated by the formation of a ladder upwards and downwards the albumin molecular weight, indicating respective albumin polymerization/agglutination and fragmentation, however not affecting the albumin levels (n=4) **(B)**. Statistical analysis performed using One-way ANOVA test.

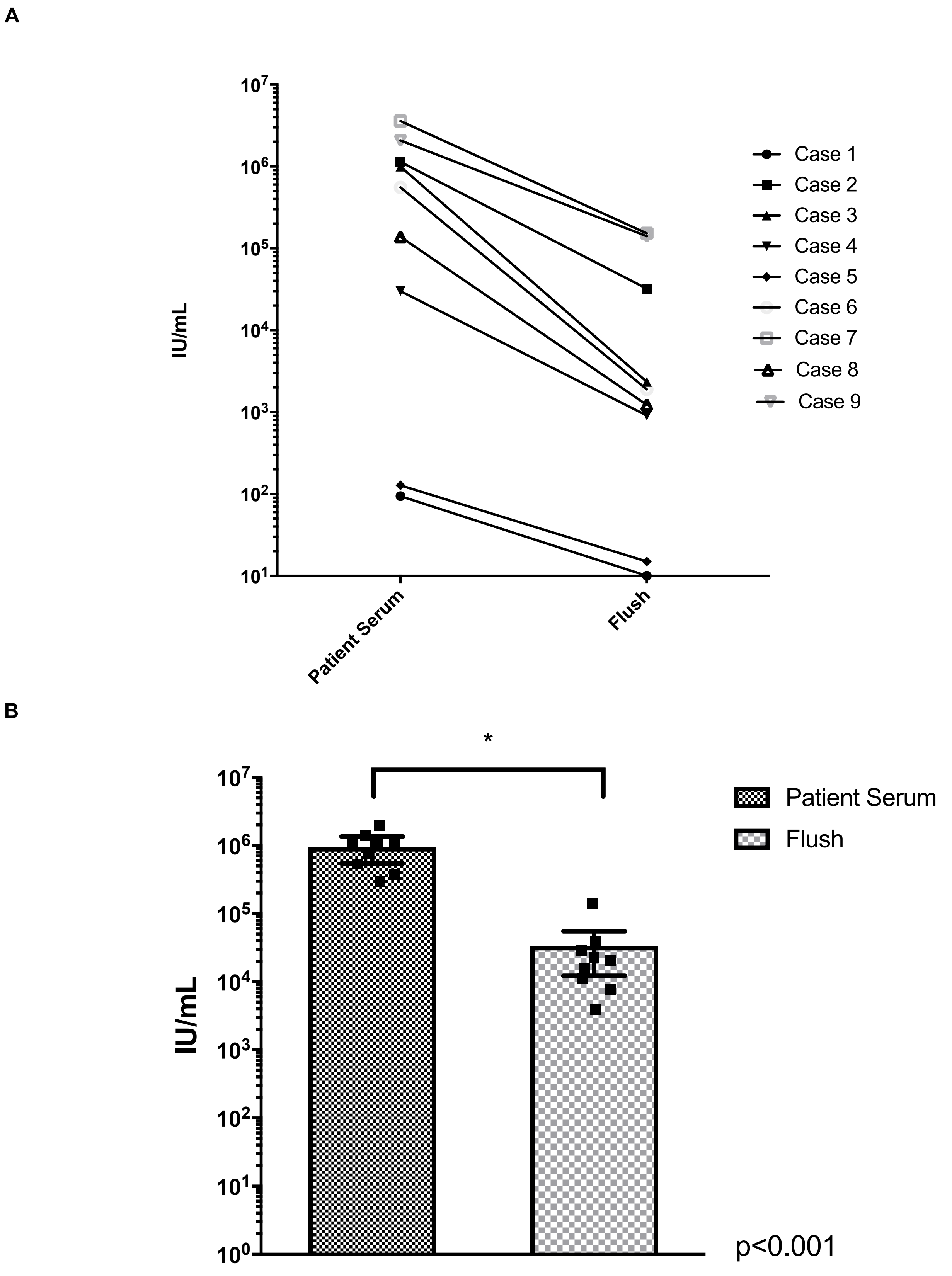

Supplementary Figure 2. Effect of cold flush on HCV levels. Upon arrival, the lung block was flushed on the back table, with Perfadex® (XVIVO), in a retrograde fashion. Samples from the flushing solution were taken from the PA and analyzed with RealTime® qPCR. **(A)** When compared to donor viremia, the flushing solution taken from the lung demonstrated an average of 1 log viral load decrease, in all the cases (n=9). **(B)** Results expressed as mean ±standard deviation of the mean (n=9). Statistical analysis performed using unpaired t-test.

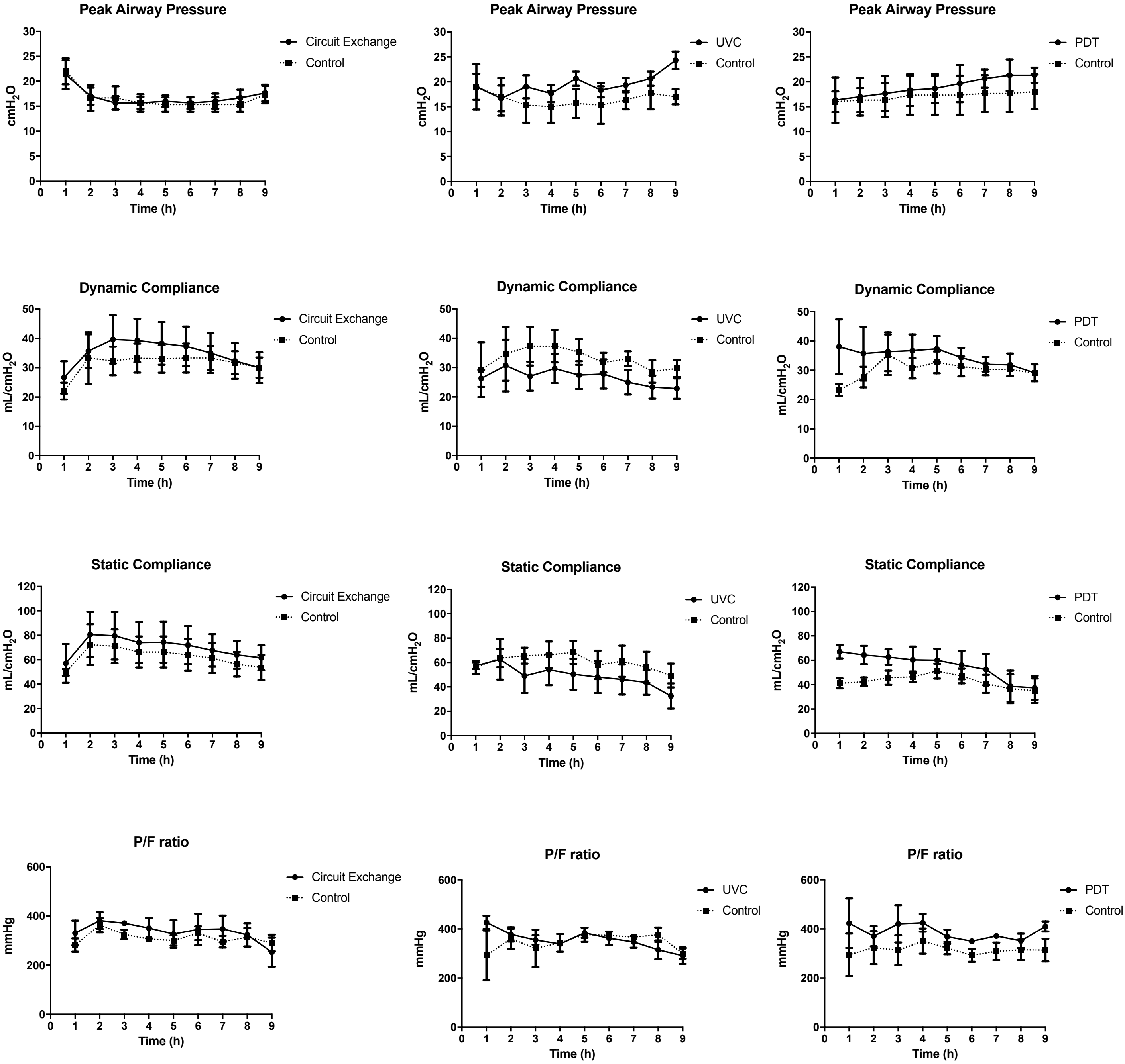

Supplementary Figure 3. Lung physiologic parameters assessed during the human HCV NAT+ EVLP phase. No statistical difference was detected in terms of Peak Airway Pressure, Dynamic Compliance, Static Compliance and oxygenation (P/F ratio) between groups, suggesting no immediate deleterious effect of UVC or PDT applied to the circuit (n=3 per group). Results are presented as mean ± standard deviation. Statistical analysis performed using One-way ANOVA test.

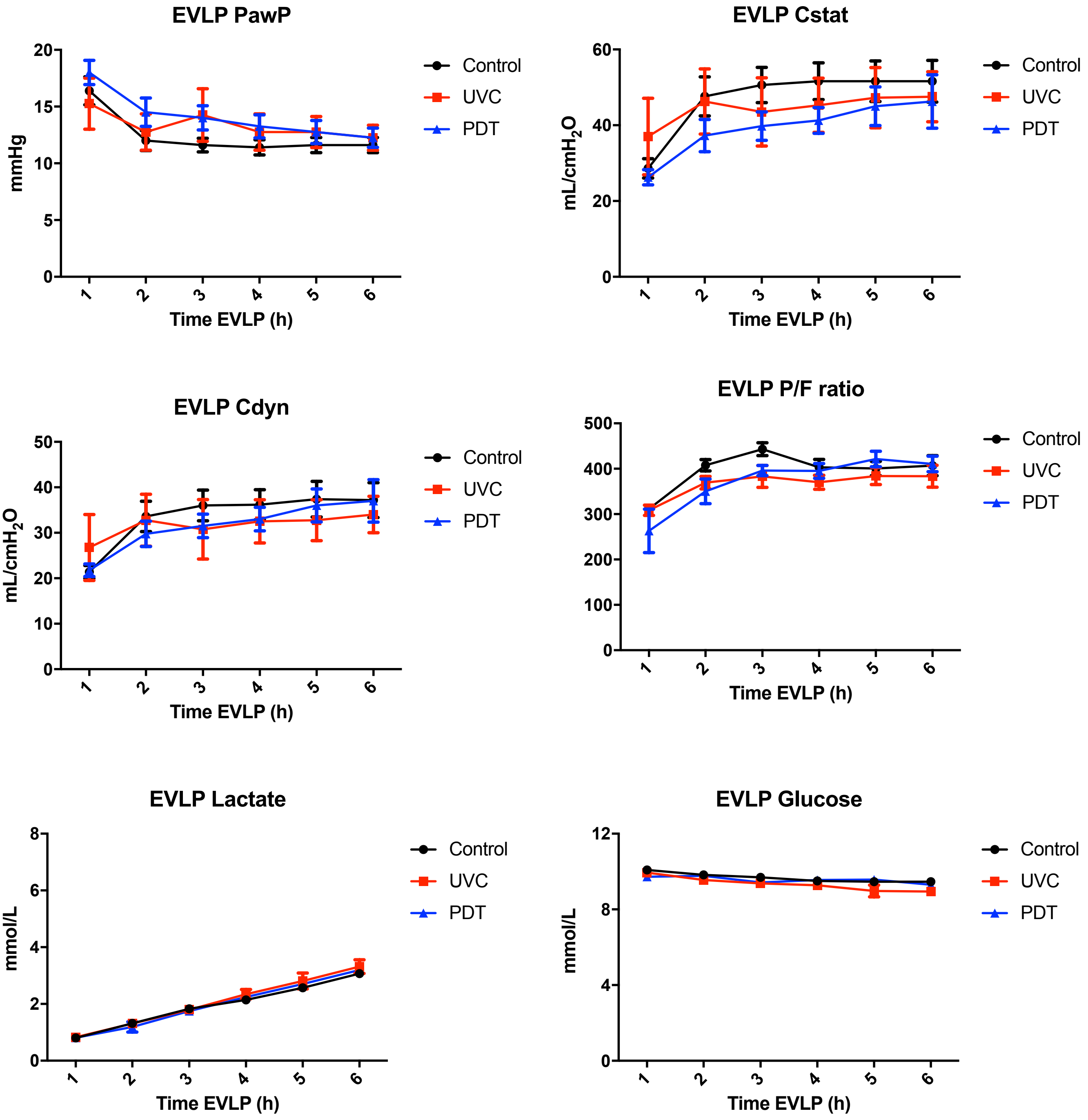

Supplementary figure 4. Large animal lung physiologic parameters assessed during the pre-clinical safety studies using EVLP/LbT treatments. No statistical difference was detected in terms of Peak Airway Pressure (PawP), Static Compliance (Cstat), Dynamic Compliance (Cdyn), Glucose, Lactate and and oxygenation (P/F ratio) between groups, suggesting no immediate deleterious effect of UVC or PDT applied to the circuit (n= 4 per group). Results are presented as mean ± standard deviation. Statistical analysis performed using One-way ANOVA test.

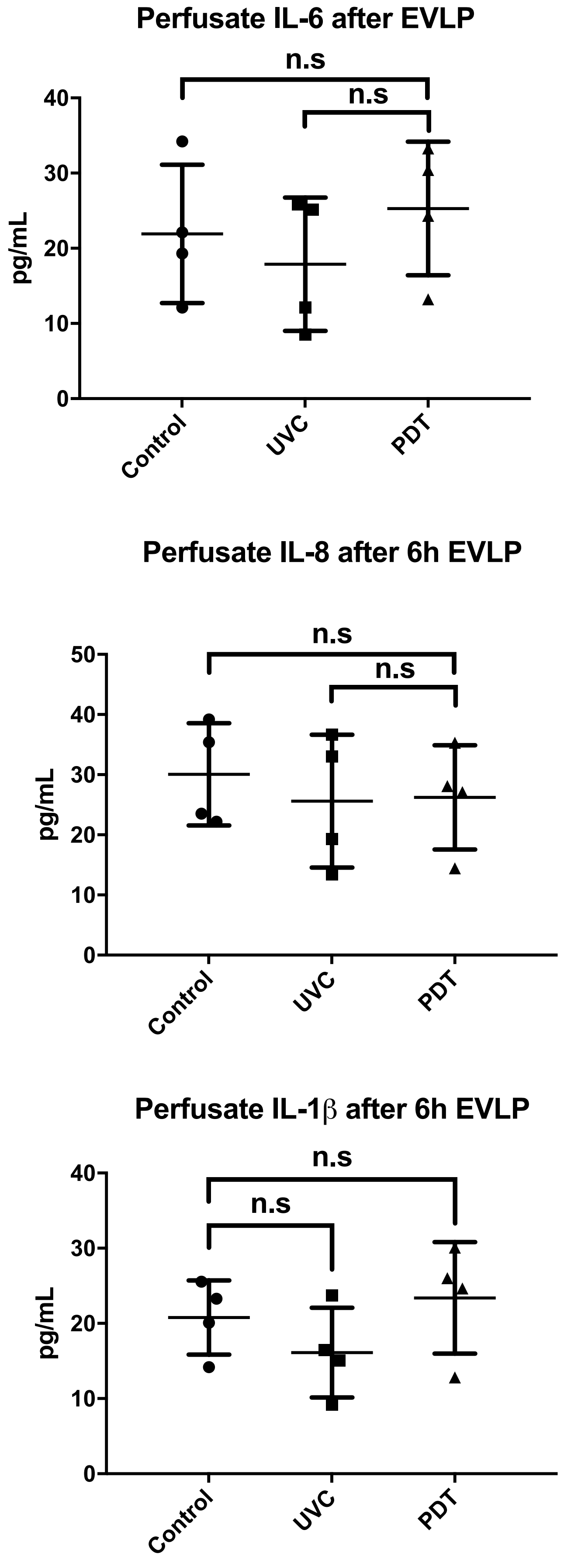

Supplementary figure 5. Pre-clinical large animal safety studies using EVLP/LbT treatments. Graft Inflammatory cytokine assessment in perfusate after 6h EVLP. No significant difference was found in perfusate IL-6, IL-8 and IL-1beta. Statistical analysis performed using One-way ANOVA test. Centre line represents mean and bounds represent standard deviation.
