# Supplementary material for: Inactivating hepatitis C virus in donor lungs using light therapies during normothermic ex vivo lung perfusion
Source: Nat Commun. 2019 Jan 29;10:481. doi: 10.1038/s41467-018-08261-z (PMC6351537; doi:10.1038/s41467-018-08261-z)
Supplement: Supplementary file 2 — Description of Additional Supplementary Files [file 41467_2018_8261_MOESM2_ESM.pdf]

Description of Additional Supplementary Files

File Name: Supplementary Movie 1
Description: The Toronto EVLP system and the customized illumination device. The device is being used to irradiate the perfusate during EVLP with UVC or red light, depending on the strategy. The apparatus was designed be part of the circuitry, in parallel with other components, without changing the pulmonary vessels pressures, also not forming air bubbles. Since the EVLP system is closed the perfusate is irradiated several times, resulting in a HCV viral particles are inactivation in a cumulative manner.

File Name: Supplementary Movie 2
Description: Delivery of Methylene Blue (1µM) to a human HCV NAT+ single lung during ex vivo lung perfusion. The photosensitizer is injected in the circuit in the hard shell reservoir, being pumped to the organ. The video depicts the moment the Methylene Blue reaches the lung, entering the organ via the pulmonary artery cannula and being drained by the left atrium cannula, also adding a blueish appearance to the perfusion solution. Video is 2x fast forwarded for demonstration purposes.
