# Supplementary material for: Inactivating hepatitis C virus in donor lungs using light therapies during normothermic ex vivo lung perfusion
Source: Nat Commun. 2019 Jan 29;10:481. doi: 10.1038/s41467-018-08261-z (PMC6351537; doi:10.1038/s41467-018-08261-z)
Supplement: Supplementary file 5 — Reporting Summary [file 41467_2018_8261_MOESM5_ESM.pdf]

nature research

Corresponding author(s): Marcelo Cypel

Reporting Summary

Nature Research wishes to improve the reproducibility of the work that we publish. This form provides structure for consistency and transparency in reporting. For further information on Nature Research policies, see Authors & Referees and the Editorial Policy Checklist.

Statistical parameters

When statistical analyses are reported, confirm that the following items are present in the relevant location (e.g. figure legend, table legend, main text, or Methods section).

n/a	Confirmed	
☐	☒	The exact sample size (*n*) for each experimental group/condition, given as a discrete number and unit of measurement
☐	☒	An indication of whether measurements were taken from distinct samples or whether the same sample was measured repeatedly
☐	☒	The statistical test(s) used AND whether they are one- or two-sided<br>*Only common tests should be described solely by name; describe more complex techniques in the Methods section.*
☐	☒	A description of all covariates tested
☒	☐	A description of any assumptions or corrections, such as tests of normality and adjustment for multiple comparisons
☐	☒	A full description of the statistics including central tendency (e.g. means) or other basic estimates (e.g. regression coefficient) AND variation (e.g. standard deviation) or associated estimates of uncertainty (e.g. confidence intervals)
☐	☒	For null hypothesis testing, the test statistic (e.g. *F*, *t*, *r*) with confidence intervals, effect sizes, degrees of freedom and *P* value noted<br>*Give P values as exact values whenever suitable.*
☒	☐	For Bayesian analysis, information on the choice of priors and Markov chain Monte Carlo settings
☒	☐	For hierarchical and complex designs, identification of the appropriate level for tests and full reporting of outcomes
☒	☐	Estimates of effect sizes (e.g. Cohen's *d*, Pearson's *r*), indicating how they were calculated
☐	☒	Clearly defined error bars<br>*State explicitly what error bars represent (e.g. SD, SE, CI)*

Our web collection on statistics for biologists may be useful.

Software and code

Policy information about availability of computer code

Data collection	Data was collected in excel files and then transferred to Graphpad prism software version 7.0
Data analysis	Graphpad prism software version 7.0

For manuscripts utilizing custom algorithms or software that are central to the research but not yet described in published literature, software must be made available to editors/reviewers upon request. We strongly encourage code deposition in a community repository (e.g. GitHub). See the Nature Research guidelines for submitting code & software for further information.

Data

Policy information about availability of data

All manuscripts must include a data availability statement. This statement should provide the following information, where applicable:

- Accession codes, unique identifiers, or web links for publicly available datasets
- A list of figures that have associated raw data
- A description of any restrictions on data availability

The datasets generated during and/or analysed during the current study are available from the corresponding author on reasonable request

Field-specific reporting

Please select the best fit for your research. If you are not sure, read the appropriate sections before making your selection.

☒ Life sciences ☐ Behavioural & social sciences ☐ Ecological, evolutionary & environmental sciences

For a reference copy of the document with all sections, see nature.com/authors/policies/ReportingSummary-flat.pdf

Life sciences study design

All studies must disclose on these points even when the disclosure is negative.

Sample size	No statistical calculations for sample sizes were performed a priori. A number of 3 per group was selected for the rejected human lung studies since each treatment lung had its own control done simultaneously and from same donor (ideal control for the study purposes). Given the reproducibility of the findings and the difficulty in obtaining such valuable organs for research we felt this was an appropriate number to determine effect of light therapies to HCV during organ perfusion as well as to obtain preliminary data on safety, especially considering additional large animal lung transplants with subsequent transplantation were performed in the phase 3 of this study. A total of 8 large animals per group (4 donors and 4 recipients) were used per group which is typical for lung injury assessments in lung transplantation studies.
Data exclusions	No data were excluded
Replication	In all phases of this study we had at least 3 replicates per group. In addition, controls were performed simultaneously in both declined human lung experiments and ex vivo mini-circuit experiments. In the large animal safety studies, 8 animals per group were (4 donors and 4 recipients) used and results were quite reproducible in regards to lung function before and after transplantation.
Randomization	Randomization was performed for human lung experiments where right or left lungs from same donor were randomly allocated to treatment or controls. In the large animal studies, randomization was also performed as per treatment to be applied or control.
Blinding	Blinding was not possible since different devices had to be added to perfusion apparatus (perfusion irradiator) as well as to perfusion solution (i.e. Methylene Blue) that the group of study would become obvious to the investigator. However, many of the assessments of samples collected such as histology, TUNEL, infectivity assays and cytokines were performed by study investigators not aware of the sample group of origin.

Reporting for specific materials, systems and methods

Materials & experimental systems

n/a	Involved in the study	
☐	☒	Unique biological materials
☐	☒	Antibodies
☒	☐	Eukaryotic cell lines
☒	☐	Palaeontology
☐	☒	Animals and other organisms
☐	☒	Human research participants

Methods

n/a	Involved in the study	
☒	☐	ChIP-seq
☒	☐	Flow cytometry
☒	☐	MRI-based neuroimaging

Unique biological materials

Policy information about availability of materials

Obtaining unique materials	No restriction exists in regards to materials used in this study. The ex vivo lung perfusion circuit and perfusate solution are commercially available by a third-part company Xvivo perfusion inc. The light irradiator device is described in detail in the methods section. The methods used for ex vivo lung perfusion and lung transplantation are described in detail in the manuscript and references. The JHF-1 virus is available commercially and references are provided.

Antibodies

Antibodies used	ELISA kits for porcine interleukin IL-1β (DY6226, IL-1 beta Pig ELISA Kit, R&D Systems, Minneapolis, MN), IL-6 (P6000B, Porcine IL-6 Immunoassay, R&D Systems, Minneapolis, MN), and IL-8 (P8000, IL-8 beta Pig ELISA Kit, R&D Systems) according to the manufacturers' instructions. TUNEL: deoxynucleotide transferase-mediated deoxy uridine triphosphate nick-end labeling: In Situ Cell Death Detection Kit, POD; Roche Diagnostics GmbH, Mannheim, Germany

	Anti-HCV core antibody: Anti-Hepatitis C Virus Core antibody (ab58713) Abcam
Validation	These are well validated antibodies noted on company websites.

Animals and other organisms

Policy information about studies involving animals; ARRIVE guidelines recommended for reporting animal research

Laboratory animals	35 kg yorkshire pigs
Wild animals	the study did not involve wild animals
Field-collected samples	this study did not involve samples collected from the field.

Human research participants

Policy information about studies involving human research participants

Population characteristics	Lungs from multi-organ donors in US where lungs were declined for clinical transplantation due to NAT+ HCV status were offered to our research studies from consented donors via IIAM (http://www.iiam.org) .
Recruitment	see above.
